# Socioeconomic disparities and infancy growth trajectory: a population-based and longitudinal study

**DOI:** 10.1186/s12887-021-02995-4

**Published:** 2021-12-04

**Authors:** Zi-yu Shao, Peng Wang, Pei Li, Yu Sun, Pei-pei Li, Peng Zhu

**Affiliations:** 1 Hefei City Maternal and Child Health & Family Planning Service Center, Hefei, China; 2 Department of Maternal, Child & Adolescent Health, School of Public Health, Anhui Medical University, Hefei, China

**Keywords:** Socioeconomic status, Body mass index, Growth, Low birth weight

## Abstract

**Background:**

The association of low socioeconomic status (SES) with childhood and adolescent obesity has been reported. It is unknown whether low SES affects body mass index (BMI) growth trajectory in the first 12 mo of life. Moreover, accelerated growth as a compensatory mechanism for low birth weight (LBW) during infancy, is an important predictor of later obesity. The aim of the present study was to examine the association of low SES with infancy BMI growth rate and trajectory for LBW and normal birth weight (NBW) infants.

**Methods:**

From September 2012 to October 2014, a total of 387 infants in this longitudinal study was subjected to repeated measures of weight and length from birth to 12 mo in Hefei. Generalized growth mixture modeling was used to classify the infancy BMI growth trajectories. Differences in infancy BMI z score (zBMI) and BMI growth rate between low SES and high SES were estimated based on linear regression after adjusting for several confounders including maternal age, pregnancy BMI, physical activity during pregnancy, paternal BMI as well as alcohol use, paternal smoking status, breastfeeding duration and delivery mode.

**Results:**

Infancy BMI trajectories in this study were classified into three categories: rapid growth (class 1), normal growth (class 2) and slow growth (class 3). Low SES infants had the higher zBMI than high SES infants for LBW group at age 6 mo [zBMI difference with 95% CI at 6 mo: 0.28(0.03, 0.53); at 12 mo: 0.21(0.01, 0.43)]. Low SES infants had more rapid zBMI growth rate than those with high SES for low birth weight between 0 and 6 months. Controlling for the confounders, these associations remained robust. We found the lower SES in the rapid growth group.

**Conclusions:**

These findings highlighted the impact of low SES on increasing BMI and accelerated growth during early infancy. Health care and relatively optimal family environment in the first 12 mo of life, especially for LBW infants, are benefit to shape the better infancy growth trajectory.

**Supplementary Information:**

The online version contains supplementary material available at 10.1186/s12887-021-02995-4.

## Introduction

Childhood overweight and obesity, an epidemic increasing public concerns, are related to poorer mental and physical health outcomes in later life [1]. Infancy body mass index (BMI) has been shown to be positively associated with later adiposity status [2, 3]. Moreover, rapid and catch-up growth during infancy are direct predictors of childhood obesity risk [3]. The Uppsala Family Study has demonstrated that higher BMI at BMI peak in the first year of life was likely to lead to higher BMI during childhood [4]. These data suggested the predicted role of the infancy BMI trajectory in BMI growth in childhood as well as adulthood. A systematic review indicated the potentially effective interventions on childhood obesity from conception to 24 mo of age [5]. It is necessary to examine the contribution of early-life environmental factors to the infancy BMI trajectory.

Previous studies found several early-life risks for accelerated growth during infancy including maternal BMI, maternal age and birth weight [6–8]. In addition, recent evidence showed that children with breastfeeding duration < 6 months had consistently higher mean BMIz from 3 to 60 months than those with breastfeeding duration ≥6 months [9]. On the other hand, paternal life-style factors, such as alcohol use and smoking status, were also linked to the infant growth such as birth weight and body fat [10]. Therefore, both maternal and paternal factors could exert the impacts on the infancy BMI trajectory.

The American Academy of Pediatrics has underlined that poverty and related social determinants of health can result in adverse health outcomes in childhood, negatively affecting physical health, socioemotional development, and educational achievement [11]. Particularly, low socioeconomic status (SES) is a robust indicator of BMI and obesity risk across the life course. The Peers and Wellness study (PAWS) found that children (mean age, 10 years) with the lower SES exhibited the higher BMI z scores (zBMI) and skinfold thickness [12]. The negatively linear associations of family SES with zBMI and adiposity in childhood were also demonstrated. Also, results from a longitudinal study in China indicated that lower family income was correlated with overweight/obesity for urban adolescents [13]. Increasing evidence has reported the diverse mechanisms in which this association operated such as low birth weight (LBW) and lack of access to health care [14]. However, these studies did not investigate the role of SES in the infancy (0-12 mo) BMI trajectory, a critical time point for monitoring growth rate.

The present study aimed to determine the impacts of family SES on infants age- and sex-standardized zBMI growth during the first 12 mo of life. Of note, catch-up growth was considered as a compensatory mechanism for LBW during infancy and LBW was correlated to childhood obesity and glucose metabolism [15]. We focused on the differences of BMI trajectory between normal birth weight (NBW) and LBW infants with socioeconomic disparities.

## Methods

### Study population

From September 2012 to October 2014, we conducted the population-based, longitudinal study. A total of 435 infants were recruited from 4 centers in Hefei including Maternal and Child Health and Family Planning Service Center of *Baohe* District, *Shushan* District, *Yaohai* District and *Luyang* District. The follow-up visits were conducted on a monthly basis and anthropometric data were collected at 0 (at birth), 1, 3, 6, 9 and 12 mo of age. Eligibility criteria of the present study included: pregnant women aged ≥18 y, lived in the urban area of Hefei, had no communication difficulties, and delivered a singleton live birth (gestational week ≥28 weeks) without assisted reproductive technology. We excluded mothers if they were diagnosed with liver, renal or thyroid dysfunction, pre-existing diabetes or anemia, gestational hypertension and gestational diabetes mellitus (*n* = 2). Moreover, 46 infants were excluded due to congenital anomalies (*n* = 15) or neonatal intensive care for 72 h (*n* = 7) or missing information on weight, length/height (*n* = 8) or loss to follow-up at 12 months (*n* = 16). In the present study, 387 infants were included for the analysis.

At the first follow-up after birth, a baseline survey of the infants’ parents was conducted by trained research personnel and the parents provided demographic information via standardized questionnaires. Maternal age at conception was recorded in years. Prepregnancy and paternal BMI were calculated from self-reported height and weight at the visit. Frequencies of moderate physical activity (such as table tennis, badminton, vigorous walking, etc.) during pregnancy were evaluated by the International Physical Activity Questionnaire (IPAQ) and dichotomized to < 30 min or ≥ 30 min per day [16]. Paternal smoking and achohol use were documented with two categories (yes or no). Socioeconomic status was assessed on the basis of the household factors (family income) as well as parental education, and classified into low and high status as previously described [12]. The duration of breastfeeding was assigned to < 6 mo and ≥ 6 mo. The characteristics of pregnant women and infants at birth was obtained from electronic medical records including gestational week, sex (male/female) and delivery mode (vaginal delivery /cesarean section). LBW, the study’s exposure variable, was defined as weighing less than 2500 g at birth and NBW was termed as weighing ≥2500 g and < 4000 g.

### Anthropometric outcomes

Anthropometrics measurements for the infants were conducted at 1, 3, 6, 9 and 12 mo in the present study. The infants were weighed to the nearest 0.01 kg on a calibrated digital scale and length/height was measured to the nearest 0.1 cm using a calibrated measuring mat or portable stadiometer [17]. BMI was calculated by dividing weight (kilograms) by height (meters) squared. BMI measurement were converted to z scores (zBMI) by using 2006 WHO standards (0–59 mo) based on the age- and sex-standardized reference data [18, 19]. We restricted the analysis to the zBMI values within ±3 SD units of the outlying zBMI. If the zBMI values were more than ±3 SD units, the values were set to missing. In the present study, inter-observer and intra-observer reliability were estimated by intra-class correlation coefficient (ICC) and ICC were 0.799.

### Confounding variables

Covariate selection was based on a backward selection procedure and other potential confounders identified in the literature [20]. The potential confounding variables were categorized into parental and infantile information. Parental information included maternal-related data such as maternal age at conception, prepregnancy BMI, education and physical activity during pregnancy, and paternal-related data such as education, BMI, income and the status of alcohol use as well as smoking status. In terms of infant’s information, gestational age, mode of delivery, birth weight, length and the duration of breastfeeding was considered.

### Statistical analysis

The characteristics of the infants were described with means ± SDs for continuous variables and counts (frequencies) for categorical variables. Analysis of variance (ANOVA) or Mann–Whitney U tests were used to compared the differences between groups for continuous or categorical data. We used repeated-measures ANOVA with SES status as the main effect and infants age as the repeated-measures variable, controlling for the confounders including maternal age, prepregnancy BMI, physical activity during pregnancy, maternal education, paternal BMI, paternal alcohol use and smoking status, paternal education, family income, gestational week, delivery mode and the duration of breastfeeding. Multiple linear regression modeling was applied to estimate the association of SES with infants zBMI at 6 mo and 12 mo. Model 1 did not adjust any confounders but Model 2 adjusted the same confounders as in the repeated-measures analysis. Statistical significance was assessed at the level of significance of 0.05.

We estimated the potential infancy BMI trajectory based on the latent class growth analysis [21]. In the present study, the latent growth curve model was used to identify heterogeneous developmental trajectories by estimating intra-individual (growth parameters intercept and slope) in the inter-individual (differences among subjects) growth paths. In addition, latent classes for the infancy BMI growth were identified on the basis of.

model fit indices: Akaike’s Information Criteria (AIC), Bayesian Information Criteria (BIC) and sample size adjusted BIC (ssaBIC), entropy, and *P*-value for Lo-Mendell-Rubin Test (LMRT) (Supplemental Table 1) [22]. The final model was chosen based on the various fit statistics and model considerations. More detailed information on modelling comparison indices for models with different number of latent classes were shown in Supplemental Table 1. In the fully study, the whole infants’ growth trajectory was divided into three categories: rapid growth (class 1), class 2 (normal growth) and class 3 (slow growth). All analyses were performed using SPSS version 22.0 software (IBM Corp).

## Results

Demographic characteristics for the infants was summarized in Table 1. The mean age of the mothers was 28.5 y. The proportion of maternal and paternal education less than 12 y were 29.5 and 27.4%, respectively. 10% of the infants live in households with a monthly income of less than 5000. In this study, the average gestational week and birth weight of the infant were 37.4 ± 2.9 week and 2.64 ± 0.69 kg.

**Table 1:** Demographics of study participants (*n* = 387)

Characteristic	n (%) or mean ± SD	Range (minimum to maximum)	*95% CI*
Maternal age, y	28.5 ± 3.7	21.0, 41.0	28.1, 28.3
Prepregnancy BMI, kg/m^2^	20.5 ± 2.5	15.8, 30.8	20.2, 20.7
Paternal BMI, kg/m^2^	24.1 ± 2.9	16.7, 33.9	23.8, 24.4
Physical activity < 30 min/d	165(42.6)	–	0.38, 0.48
Paternal achohol use	214(55.3)	–	0.50, 0.60
Paternal smoking status	176(45.5)	–	0.40, 0.51
Maternal Education ≤12 y	114(29.5)	–	0.66, 0.75
Paternal education ≤12 y	106(27.4)	–	0.68, 0.77
Family income < 5000 yuan/mo	95(24.5)	–	0.71, 0.80
Gestational week (week)	37.4 ± 2.9	28.0, 41.0	37.1, 37.7
Female	188(48.6)	–	0.44, 0.54
Cesarean section	219(56.6)	–	0.38, 0.49
Breastfeeding duration< 6 mo	323(83.5)	–	0.13, 0.21
Birth weight (kg)	2.64 ± 0.69	1.10, 4.00	2.59, 2.73
BMI *z* score at birth	−0.14 ± 1.38	−4.05, 3.10	−1.43, −1.16

In Model 1 and Model 2, we found that low SES was associated with the higher zBMI in the infants for LBW group at age 6 mo and 12mo [zBMI difference with 95% CI at 6 mo: 0.28(0.03, 0.53); at 12 mo: 0.21(0.01, 0.43)] (Table 2 and Supplemental Table 2). Similarly, low SES infants had more rapid zBMI growth rate than those with high SES for low birth weight between 0 and 6 months in Model 1 and Model 2. However, these significant differences were not observed among the infants in NBW group. The stratified analysis indicated that breastfeeding duration did not show the modifiable impacts on the relationship between SES and zBMI growth at age 6 mo or 12 mo (Table 3).

**Table 2:** Associations of infant SES with differences of zBMI growth separated by birth weight in each growth period

	Model 1	Model 2
Growth periods	Mean difference (95% CI) between low SES vs. high SES	
Normal birth weight		
6 mo	− 0.11(− 0.47, 0.25)	−0.20(− 0.58, 0.18)
9 mo	− 0.08(− 0.43, 0.26)	−0.19(− 0.54, 0.16)
12 mo	− 0.14(− 0.48, 0.21)	−0.25(− 0.60, 0.11)
0-6 mo	0.03(− 0.38, 0.43)	0.05(− 0.38, 0.47)
6-12 mo	− 0.03(− 0.34, 0.28)	−0.05(− 0.38, 0.28)
Low birth weight		
6 mo	0.27(0.03, 0.51)	0.28(0.03, 0.53)
9 mo	0.14(−0.05, 0.34)	0.16(−0.04, 0.37)
12 mo	0.18(0, 0.40)	0.21(0.01, 0.43)
0-6 mo	0.35(0.03, 0.66)	0.34(0.01, 0.66)
6-12 mo	−0.12(−0.34, 0.11)	−0.13(− 0.36, 0.09)

Values were expressed as the difference in low SES compared with high SES. Model 1 did not adjust any confounders. Model 2 adjusted maternal age, pregnancy BMI, physical activity during pregnancy, paternal BMI as well as alcohol use, paternal smoking status, breastfeeding duration and delivery mode

**Table 3:** The modifiable effects of breastfeeding duration on the associations of low birth weight infant SES with differences of zBMI growth

	Breastfeeding duration< 6 mo	Breastfeeding duration≥6 mo
Growth periods	Mean difference (95% CI) between low SES vs. high SES	
6 mo	0.27(− 0.01, 0.54)	0.52(− 0.17, 1.21)
9 mo	0.09(− 0.13, 0.30)	0.85(0.24, 1.46)
12 mo	0.17(−0.06, 0.39)	0.67(− 0.09, 1.43)
0-6 mo	0.29(−0.05, 0.63)	0.69(− 0.17, 1.55)
6-12 mo	−0.16(− 0.41, 0.08)	−0.02(− 0.63, 0.59)

Values were expressed as the difference in low SES compared with high SES, adjusting maternal age, pregnancy BMI, physical activity during pregnancy, paternal BMI as well as alcohol use, paternal smoking status and delivery mode

**Fig. 1:**
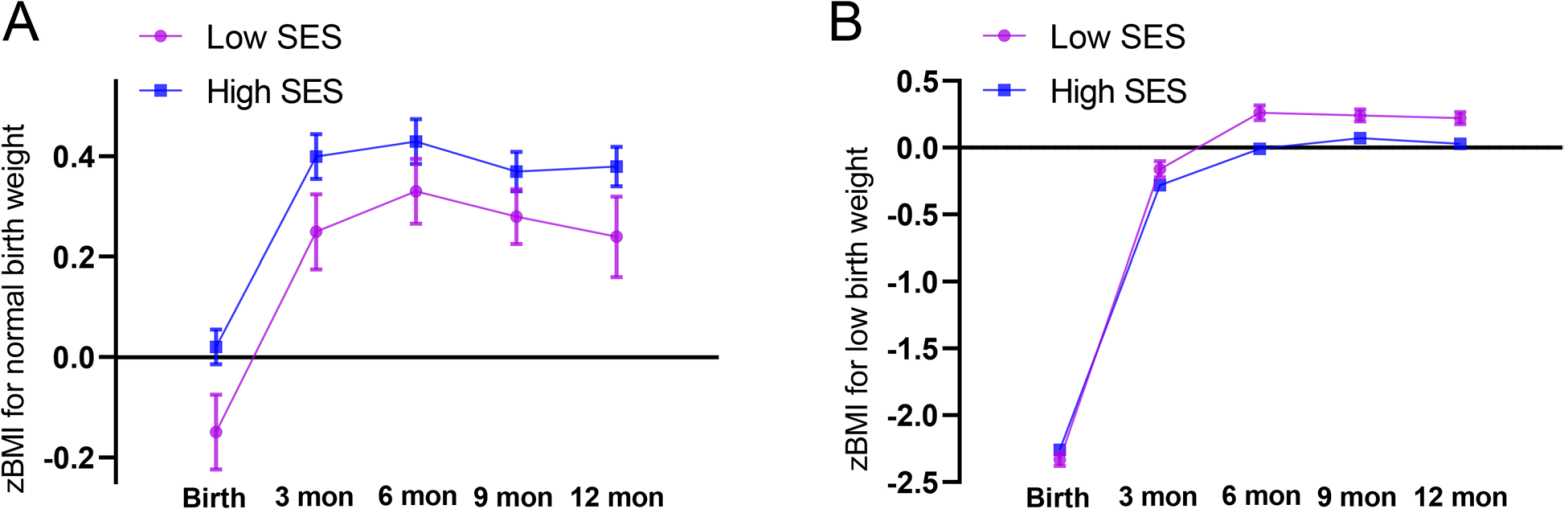
Trajectories of BMI z scores in high and low SES separated by normal birth weight and low birth weight. Results are shown with mean z scores ± SEMs. Repeated-measures ANOVA adjusted for maternal age, pregnancy BMI, physical exercise during pregnancy, paternal BMI as well as alcohol use, paternal smoking status, breastfeeding duration, and delivery mode in **A** and **B**. Trajectories in BMI z scores displayed no significant differences between high and low SES for normal birth weight (*P* = 0.370) and low birth weight (*P* = 0.263)

**Fig. 2:**
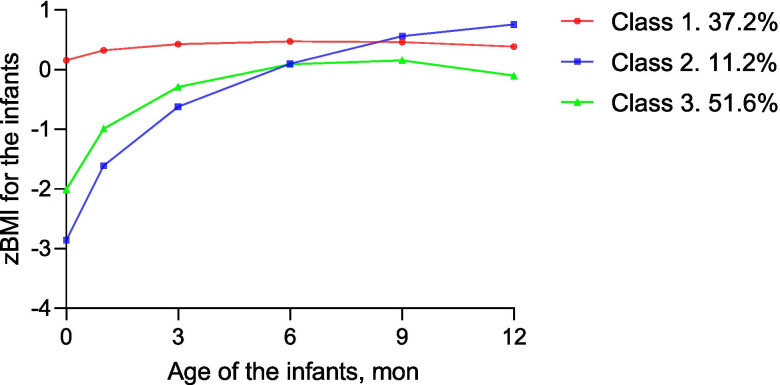
Heterogeneous developmental trajectories for the full sample

The trajectories of infancy zBMI in socioeconomic disparities by birth weight from birth to 12 mo were exhibited in the Supplemental Table 3 and Fig. 1. After adjusting a series of confounders, results from the repeated-measures ANOVA showed no significant differences of zBMI between low SES and high SES group for the infants of LBW (*P* = 0.370) or NBW (*P* = 0.263). Nonetheless, compared with the infants with high SES, those with low SES displayed the increasing zBMI especially in 6 mo and 12 mo (zBMI for 6 mo: 0.26 ± 0.11 for low SES VS − 0.01 ± 0.07 for high SES; zBMI for 6 mo 12 mo: 0.22 ± 0.09 for low SES VS 0.03 ± 0.07 for high SES). Infancy BMI growth trajectory by the latent growth curve model was divided into three categories: rapid growth (class 1), normal growth (class 2) and slow growth (class 3) via latent growth curve modeling (Fig. 2). The description of characteristics among 3 groups were shown in Table 4. In the rapid growth group, the proportion of the infants with family income less than 5000 yuan (17.9%) per month were higher (*P* = 0.042). The infants in the normal growth group had higher gestational week (38.0 ± 2.4 week), birth weight (2.81 ± 0.70 kg) and BMI z score at birth (− 1.06 ± 1.34) (*P* < 0.001).

**Table 4:** Demographics of the infants with heterogeneous developmental trajectories

Characteristic	Rapid (*n* = 39)	Normal (*n* = 144)	Slow (*n* = 204)	*P*
	n (%) or mean ± SD			
Maternal age, y	28.9 ± 4.0	28.9 ± 3.8	28.1 ± 3.5	0.126
Prepregnancy BMI, kg/m^2^	20.2 ± 2.5	20.7 ± 2.6	20.3 ± 2.2	0.231
Paternal BMI, kg/m^2^	24.2 ± 2.6	24.4 ± 2.7	23.8 ± 3.0	0.249
Physical activity < 30 min/d	19(48.7)	65(45.1)	81(39.7)	0.433
Paternal achohol use	27(69.2)	80(55.6)	107(52.5)	0.154
Paternal smoking status	21(53.8)	71(49.3)	84(41.2)	0.176
Maternal Education ≤12 y	15(38.5)	44(30.6)	55(27.0)	0.330
Paternal education ≤12 y	12(30.8)	43(29.9)	51(25.0)	0.535
Family income < 5000 yuan/mo	7(17.9)	10(6.9)	22(10.8)	**0.042**
Gestational week (week)	34.9 ± 3.9	38.0 ± 2.4	37.4 ± 2.8	**< 0.001**
Female	21(53.8)	78(54.2)	89(43.6)	0.120
Cesarean section	26(66.7)	80(55.6)	113(55.4)	0.408
Breastfeeding duration< 6 mo	31(79.5)	124(86.1)	168(82.4)	0.507
Birth weight, kg	2.22 ± 0.66	2.81 ± 0.70	2.61 ± 0.66	**< 0.001**
BMI *z* score at birth	−2.02 ± 1.28	−1.06 ± 1.34	−1.47 ± 1.38	**< 0.001**

Together, this study found the association of low SES with the higher zBMI in the infants for LBW group at age 6 mo and 12mo. Moreover, the infants with low SES had more rapid zBMI growth rate than those with high SES for low birth weight between 0 and 6 mon in LBW group. The trajectories of infancy zBMI for them were characterized as the accelerated growth.

## Discussion

The aim of present longitudinal study was to estimate the association of family SES with the infancy BMI growth. We observed the lower SES in the infants with rapid growth rate and LBW infants had the higher zBMI growth rate from 6 mo and 12mo if they were low SES. After adjusting for the cofounding variables, our results suggested that low SES infants had more rapid zBMI growth than high SES infants for LBW but not for normal weight from birth to 6 mo. Exclusively breastfeeding more than 6 mo did not modify the association of SES with BMI growth rate during infancy. Our findings indicated that low SES was associated with increasing zBMI growth for the infants with LBW during the first 12 mo of life.

Infants who were at the high BMI or accelerated growth during infancy were at the high risks for later obesity [2]. We found that low SES was related to the higher zBMI, which emphasized the low SES as a risk factor of subsequent obesity. Therefore, the association of low SES with childhood or adolescent obesity was possibly mediated by rapid BMI growth in the first 12 mo of life. Results from PAWS shown that low SES families had the highest zBMI during children’s kindergarten year [12]. Another recent longitudinal study in China also found that the adolescent overweight/obesity could be predicted by low family income in urban but not for rural China [13]. However, these two studies did not examine the relationship between SES and zBMI growth in early infancy, a critical time window for intervention for childhood obesity prevention. Evidence on investigating the role of SES in the BMI growth trajectory was limited. In the present study, low SES among LBW infants was associated with higher zBMI at 6 mo as well as higher zBMI growth rate between 0 and 6 months. Increasing BMI growth rate could further lead to excessive adiposity rebound and higher BMI at early infancy [3]. We did not observe the specific modifiable impact of breastfeeding duration on the association between low SES and BMI growth, which emphasized the independent role of SES in the BMI. Together, these results showed that the onset of childhood and adolescent obesity in the low SES families could occur in the infancy.

We found that infants with NBW had the higher zBMI in comparison with LBW infants from birth to 12 mo while higher zBMI growth rate were among LBW infants between birth and 6 mo. Results from a prospective cohort study were consistent with ours, which extended the infants aged 1-18 mo [6]. Further, in term of LBW infants, we observed the higher zBMI and more rapid growth rate in low SES infants. Low SES was linked to multiple risk factors such as parental stress, unhealthy diet, poor physical activity behaviors, noise and pollutant [23]. An early review has documented the association of low SES with increasing BMI, adiposity and obesity possibly mediated by LBW [14]. Another representative nation-wide survey in Germany indicated that LBW partially mediated the association of maternal smoking in pregnancy and higher BMI in children and adolescents aged 3–17 y [24]. Therefore, our findings suggested that the infants exposed to dual stressing including low SES and LBW were more inclined to have higher zBMI and BMI growth rates.

Our study has several strengths. First, multiple time point of monitoring infant growth in the first 12 mo of life was applied in this longitudinal design. Second, this is the first to focus on the impacts of low SES on the infancy BMI growth of NBW infants with those with LBW. Finally, we adjusted for variables in the relation between SES and zBMI trajectories during the first year including maternal lifestyle (physical activities during pregnancy) and the infants’ characteristics such as duration of breastfeeding.

One limitation was the small sample size of the present study, which resulted in the potential selection bias and statistical power reduction. No causal inferences could be determined in that this is an observational study. Considering this investigation in a single center, our research findings needed be verified in diverse ethnic groups.

## Conclusion

In summary, we observed the higher BMI growth rate in low SES infants in the first of 12 mo. For LBW infants, low SES was significant associated with higher zBMI at age 6 mo as well as 12 mo and BMI growth rate in the first 6 mo of life. Our results implied that the impact of low SES on increasing BMI and accelerated growth during early infancy. These findings indicated that poverty in urban areas should be a potential predictor of the later obesity in other districts. Although the previous study has reported the relationship between low SES and childhood obesity, future research needs to explore the impacts of socioeconomic environments on human development among children who underwent adverse pregnant outcomes such as premature delivery and LBW [12]. Health care and relatively optimal family environment in the prenatal and early postnatal period, especially for LBW infants, are essential to establish the better growth trajectory for preventing and intervening the later overweight or obesity.

## Supplementary Information

**Additional file 1: Supplemental Table 1.** Model comparison indices for models withdifferent number of latent classes. **Supplemental Table 2.** Regressioncoefficients (95% confidence intervals) of predictorsforBMI z scores in infants with low birth weight in Model 2. **Supplemental Table 3.** Trajectoriesof BMI z scores in high and low SES separated by normal birth weightand low birth weight.

## Data Availability

The datasets used and/or analyzed during the current study are available from the corresponding author on reasonable request.

## Abbreviations

BMI: Body mass index
SES: Socioeconomic status
zBMI: BMI z score
LBW: Low birth weight
NBW: Normal birth weight
