# Supplementary material for: Socioeconomic disparities and infancy growth trajectory: a population-based and longitudinal study
Source: BMC Pediatr. 2021 Dec 4;21:549. doi: 10.1186/s12887-021-02995-4 (PMC8642984; doi:10.1186/s12887-021-02995-4)
Supplement: Supplementary file 1 — Additional file 1: Supplemental Table 1. Model comparison indices for models withdifferent number of latent classes. Supplemental Table 2. Regressioncoefficients (95% confidence intervals) of predictorsforBMI z scores in infants with low birth weight in Model 2. Supplemental Table 3. Trajectoriesof BMI z scores in high and low SES separated by normal birth weightand low birth weight. [file 12887_2021_2995_MOESM1_ESM.docx]

Supplemental Table 1. Model comparison indices for models with different number of latent classes.

| Class | AIC | BIC | aBIC | Entropy | LMRT | Log likelihood |
| --- | --- | --- | --- | --- | --- | --- |
| 1 | 5625.05 | 5687.43 | 5639.84 |  |  |  |
| 2 | 5570.95 | 5646.16 | 5585.88 | 0.83 | <0.001 | -2799.03 |
| 3 | 5559.52 | 5650.56 | 5577.59 | 0.79 | 0.039 | -2789.65 |
| 4 | 5551.04 | 5657.91 | 5572.24 | 0.84 | 0.219 | -2756.76 |

ABIC, adjusted BIC; BIC, Bayesian information criteria, LMRT, *P*-value for Lo-Mendell-Rubin Test

Supplemental Table 2. Regression coefficients (95% confidence intervals) of predictors

for BMI z scores in infants with low birth weight in Model 2.

| Predictor variable | zBMI at 6 mo | zBMI at 9 mo | zBMI at 12 mo |
| --- | --- | --- | --- |
| Low SES | 0.28(0.03, 0.53) | 0.16(-0.04, 0.37) | 0.21(0.01, 0.43) |
| Maternal age, y | -0.01(-0.04, 0.02) | 0(-0.03, 0.03) | -0.01(-0.04, 0.02) |
| Prepregnancy BMI, kg/m^2^ | 0.03(-0.01, 0.08) | 0.04(-0.01, 0.08) | 0.04(0, 0.09) |
| Paternal BMI, kg/m^2^ | 0.02(-0.02, 0.06) | 0.02(-0.02, 0.06) | 0.01(-0.03, 0.05) |
| Physical activity <30min/d | 0.02(-0.22, 0.25) | 0(-0.22, 0.22) | -0.12(-0.35, 0.11) |
| Breastfeeding duration<6 mo | 0.24(-0.13, 0.60) | 0.22(-0.12, 0.57) | 0.17(-0.19, 0.53) |
| Cesarean section | -0.06(-0.30, 0.17) | -0.03(-0.25, 0.19) | 0.05(-0.18, 0.28) |
| Paternal achohol use | -0.07(-0.32, 0.17) | -0.05(-0.28, 0.18) | 0.07(-0.18, 0.31) |
| Paternal smoking | 0.14(-0.11, 0.38) | 0.06(-0.17, 0.29) | -0.02(-0.26, 0.22) |

SES, low socioeconomic status.

Supplemental Table 3. Trajectories of BMI z scores in high and low SES separated by normal birth weight and low birth weight.

|  | Low SES |  | High SES |
| --- | --- | --- | --- |
|  | zBMI (mean ± SEMs) | | |
| Normal birth weight |  |  |  |
| zBMI0 | -0.15±0.15 |  | 0.02±0.07 |
| zBMI1 | 0.48±0.13 |  | 0.26±0.07 |
| zBMI3 | 0.25±0.15 |  | 0.40±0.09 |
| zBMI6 | 0.33±0.13 |  | 0.43±0.09 |
| zBMI9 | 0.28±0.11 |  | 0.37±0.08 |
| zBMI12 | 0.24±0.16 |  | 0.38±0.08 |
| Low birth weight |  |  |  |
| zBMI0 | -2.33±0.10 |  | -2.26±0.06 |
| zBMI1 | -1.02±0.09 |  | -0.95±0.07 |
| zBMI3 | -0.16±0.12 |  | -0.28±0.07 |
| zBMI6 | 0.26±0.11 |  | -0.01±0.07 |
| zBMI9 | 0.24±0.09 |  | 0.07±0.07 |
| zBMI12 | 0.22±0.09 |  | 0.03±0.07 |

SES, low socioeconomic status.
